# Pilot study: validity and reliability of textile insoles used to measure the characteristics of landing tasks during rehabilitation and artistic gymnastics

**DOI:** 10.1186/s13104-023-06328-9

**Published:** 2023-04-22

**Authors:** Delia Schümperlin, Christoph Schärer, Luzia Kalberer, Stephen J. Ferguson, Silvio R. Lorenzetti

**Affiliations:** 1 ETH Zurich, Institute for Biomechanics, Hönggerbergring 64, 8093 Zurich, Switzerland; 2 Swiss Federal Institute of Sport Magglingen (SFISM), Hauptstrasse 247, 2532 Magglingen, Switzerland

**Keywords:** Ground reaction force, Impulse, Landings, Force–time curve, Statistical parametric mapping, Root mean square error, Intraclass correlation coefficient, Bland-Altmann diagrams

## Abstract

**Objectives:**

Artistic gymnastics is a sport where most athletes start at an early age and training volumes are high. Hence, overuse and acute injuries are frequent due to the load endured during landing tasks. During landing, the ground reaction force (GRF) is up to 15.8 times the body weight and therefore reliable GRF measurements are crucial. The gold standard for GRF measurements are force plates. As force plates are mostly used in a constrained laboratory environment, it is difficult to measure the GRF in representative training settings. Textile insoles (novel GmbH, Munich, Germany) exist, which can be used to measure dynamic GRF. Hence, the motivation of this study is to test the validity and reliability of these insoles during landing tasks. GRF was measured during four different exercises, in two test subjects and compared to concurrent force plate data.

**Results:**

Twelve out of 16 statistical parametric mapping plots showed no significant difference between the measured force curves of insoles and force plates. Across conditions, the root mean square error of the maximal vertical GRF was 21 N/kg and an impulse 0.4 Ns/kg. The intraclass correlation coefficient (ICC 2,1) ranged from 0.02 to 0.76 for maximal vertical GRF and from − 0.34 to 0.76 for impulse. The insoles are a valid measurement tool for GRF curve progression and impulse during landing but underestimate the maximal vertical GRF.

**Supplementary Information:**

The online version contains supplementary material available at 10.1186/s13104-023-06328-9.

## Introduction

Gymnastics is a sport with various disciplines and many people of all ages and levels practice it [1]. Most athletes start at an early age and engage in high volume training [1]. Therefore, overuse and acute injuries are prevalent, especially due to high impacts during landing [2]. The injury rate is 10.7% for female and 8.3% for male elite gymnasts during recent Olympic Games [3]. The most frequently affected body parts are the lower limbs, which account for 36–70% of all injuries [4]. Here, more than half of the lower limb injuries occur during landing, where the ground reaction force (GRF) has been measured at 7.1–15.8 times the body weight [4, 5].

To measure GRF, force plates are the gold standard for laboratory settings [4] and are producing valid data over years [6]. The transfer to a training setting is challenging, especially due to the damping materials in the gymnastics hall. As a different measurement technology, accelerometers are used to assess the acceleration of the lower limbs or other parts of the body [7]. Here, a major limitation is the fact that the results are highly influenced by the wobbling mass and the fixation of the sensors [7]. Recently, a new textile insole to measure plantar pressure based on the GRF was introduced (novel GmbH, Munich, Germany). Insoles can easily be used in different settings [8].

Therefore, the goal of this study is first to test the validity of these insoles in terms of maximal vertical GRF and impulse during different landing tasks. The second goal is to assess the reliability of the insoles. The motivation for this study is to identify a method for measuring the load caused by landing during physiotherapy exercises and gymnastics training. This information will allow the gymnast to evaluate whether the landing tolerance during rehabilitation is sufficient to execute a certain gymnastic jump respectively element.

## Main text

### Materials and methods

#### Study design and ethics

The study was conducted as an observational single-centred study. The measurements were taken during several days between May and July 2022 in Magglingen (Switzerland). In total, two recreational athletes (Participant 1: 25 years, 1.70 m, 57 kg, female and Participant 2: 24 years, 1.85 m, 90.8 kg, male) were involved in this study. Included were participants who were healthy, between 18 and 45 years, and had no acute injuries of lower extremities or lower back. This study was approved according to the guidelines of the internal review board of the Swiss Federal Institute of Sport Magglingen (SFISM). All study procedures were conducted in accordance with the Declaration of Helsinki and a written informed consent was obtained from all participants.

#### Validity and reliability measurements

Plantar pressure measurements were performed using novel loadsol^®^ pro insoles (loadsol^®^ pro medial lateral posterior, novel GmbH, Munich, Germany) with a measurement frequency of 200 Hz. The insole data were recorded on the associated application “loadsol-s” on a tablet (iPad Pro 11’’ (2021), Apple Corporation, Cupertino, CA, USA). The insoles were fixated on the soles of the feet during all measurements, because gymnasts normally train barefoot and the measurements should be specific to artistic gymnastics. First, each foot was cleaned with ethanol and the insole was then attached with elastic adhesive bandage (see Additional file 1: Fig. S1). To zero the insoles, the participants balanced on one foot, then the other. Additionally, force plates (Type Kistler 9260AA6, Kistler Instrumente AG, Winterthur, Switzerland) were used concurrently for all measurements. Measurements with Participant 1 were carried out with one force plate. For the Participant 2 an experimental setting with two force plates was used, where a left/right comparison would be possible. However, in this work the total landing force was of interest. The validity and reliability measurements included the following exercises: countermovement (CMJ), squat (SJ), drop jumps (DJ) and drop landings (DL). The DJ and DL were executed from 20, 40 and 60 cm height. Each exercise was repeated five times. In order to familiarise themselves with the exercises, all participants had at least one test run for each task.

#### Data analysis and statistics

Data analysis was conducted using spreadsheet software (Microsoft Excel 2016, Microsoft Corporation, Redmond, WA, USA), R Studio (Version 2022.02.2, R Studio, Boston, MA, USA) and MATLAB (Version R2017b, MatWorks, Natick, MA, USA). The data of the force plate(s) were downsampled to 200 Hz, allowing better comparison with the insole data. A jump was considered to be invalid when the novel loadsol^®^ pro insole measurements had multiple dropouts, which would lead to inaccurate data.

To assess the validity and reliability of the insoles, the force–time curve progression, maximal vertical GRF and impulse were analysed. The maximal vertical GRF was defined at the landing point where the left and right insole, respective to the force plate, had the highest combined value. In order to synchronise the insoles and force plate, the maximal vertical GRF was used. The impulse was calculated according to the maximal vertical GRF and only values greater than the participant’s body weight were included.

To compare the force–time curve of the two measurement technologies, the maximal vertical GRFs were superimposed and the curves were then compared in a force–time graph in Microsoft Excel. Additionally, statistical parametric mapping (SPM) was performed in MATLAB, where the analysed time interval was from 0.04 s before to 0.2 s after the maximal vertical GRF (maximal vertical GRF at 0.04 s, which means on the x-axis at x = 9) [9]. The root mean square error (RMSE), intraclass correlation coefficient (ICC 2,1) with a 95% confidence interval and Bland-Altmann diagrams of the maximal vertical GRF and impulse were generated [10–12]. The RMSE and Bland-Altmann diagram were created in Microsoft Excel. The ICC was calculated in R Studio and the following categorisation scheme was used: < 0.5 poor, 0.5–0.75 moderate, 0.75–0.9 good and > 0.9 excellent [11]. The level of statistical significance was set to p < 0.05.

### Results

**Fig. 1:**
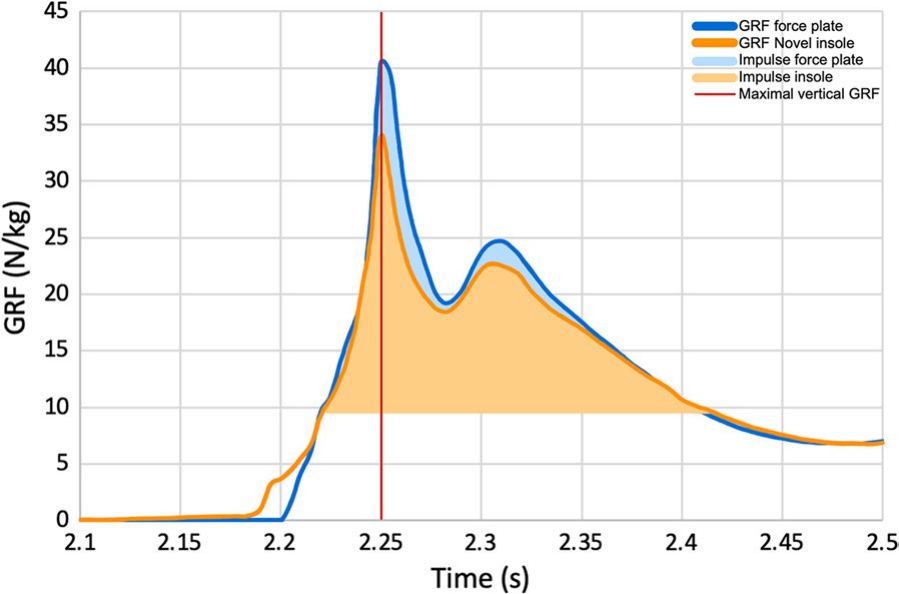
Ground reaction force (GRF) of one DL 20 cm of Participant 2. The maximal vertical GRF is marked in red. The force plate and insole measurement are labelled in dark blue, respectively dark orange. The impulse is highlighted in light blue for the force plate and light orange for the insole measurement. *DL*: drop landing

In total, 66 valid jumps were included in the analysis. A force–time graph of a single jump (Participant 2, DL 20 cm) is shown in Fig. 1, where the maximal vertical GRF and impulse are marked. In the supplement, other force–time graphs are included, demonstrating that curves are generally similar to curves of force plate measurements (see Additional file 1: Fig S2). The SPM plots of all exercises of both participants are presented in the supplement, with higher variation at the beginning (x = 0 to 15) of the analysed time frames (Additional file 1: Fig. S3). Twelve out of 16 plots demonstrated no significant difference between the insole and force plate curves. Additionally, in Table 1, the force plate and insole (mean ± standard deviation (SD)), RMSE and ICC values of maximal vertical GRF and impulse are presented. The RMSE of maximal vertical GRF deviated from the force plate values by 32% and those of impulse differed by 10%. Most ICC values for maximal vertical GRF and impulse both denoted a poor association between the novel loadsol^®^ pro insoles and force plate except for DJ 40 cm in maximal vertical GRF, CMJ and DJ 60 cm in impulse measurements. Detailed tables of all RMSE and ICC values for both variables of interest are attached in the Additional file 1: Tables S1–S4). The Bland-Altmann diagrams for maximal vertical GRF and impulse of both participants are added in the Additional file 1: Figs. S4 and S5).

**Fig. 2:**
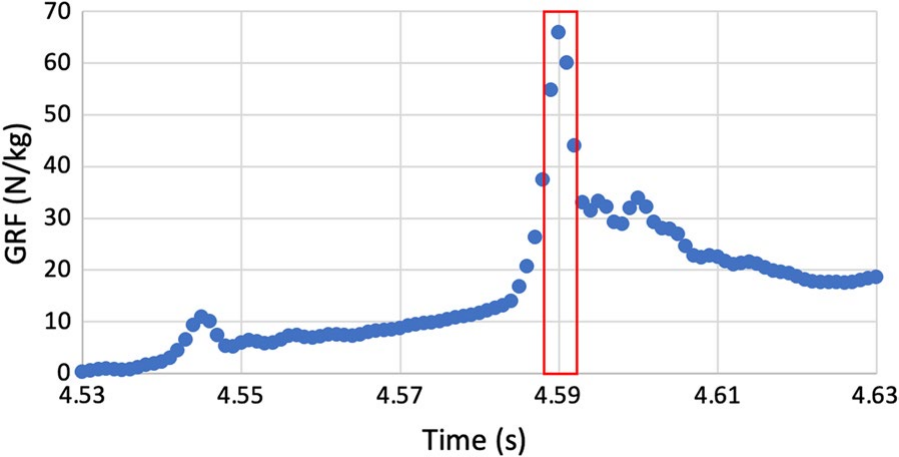
Ground reaction force (GRF) of a counter movement jump (CMJ). The maximal vertical GRF of one countermovement jump on the force plate is recorded by 1000 Hz which corresponds to five reading points (labelled in red). In contrast, the insoles have a sampling rate of 200 Hz that would lead to only one reading point in the same time period of 0.005 s. *CMJ* countermovement jump

**Table 1:** Summary of statistical analysis for maximal vertical GRF (left) and impulse (right) of both participants

	Maximal vertical GRF					Impulse				
Exercise	∅ force plate (N/kg) and SD	∅ insole (N/kg) and SD	RMSE (N/kg)	ICC	ICC 95% confidence interval	∅ force plate (Ns/kg) and SD	∅ insole (Ns/kg) and SD	RMSE (Ns/kg)	ICC	ICC 95% confidence interval
CMJ n = 10	54 ± 13	43 ± 6	17	0.23	[− 0.36, 0.85]	3.5 ± 0.2	3.6 ± 0.4	0.3	0.76	[0.05, 0.97]
SJ n = 9	68 ± 14	44 ± 3	28	0.05	[− 0.16, 0.69]	3.3 ± 0.2	3.3 ± 0.1	0.2	0.3	[− 0.57, 0.88]
DJ 20 cm n = 7	49 ± 5	41 ± 3	10	0.11	[− 0.28, 0.87]	4.4 ± 0.1	4.2 ± 0.1	0.3	0.21	[− 0.15, 0.89]
DJ 40 cm n = 8	61 ± 13	53 ± 9	11	0.76	[− 0.18, 0.98]	5.0 ± 0.1	4.9 ± 0.1	0.2	0.39	[− 0.12, 0.86]
DJ 60 cm n = 7	78 ± 9	67 ± 8	19	0.13	[− 0.56, 0.91]	5.6 ± 0.2	5.5 ± 0.2	0.2	0.73	[− 0.17, 0.99]
DL 20 cm n = 8	47 ± 10	35 ± 6	16	0.02	[− 0.69, 0.87]	2.2 ± 0.2	2.2 ± 0.3	0.4	− 0.34	[− 1.13, 0.87]
DL 40 cm n = 8	79 ± 16	46 ± 4	36	0.04	[− 0.10, 0.63]	3.3 ± 0.1	3.5 ± 0.7	0.9	0.12	[− 0.48, 0.88]
DL 60 cm n = 9	81 ± 23	50 ± 10	36	0.25	[− 0.14, 0.86]	3.9 ± 0.1	3.5 ± 0.3	0.5	0.12	[− 0.22, 0.78]
Mean (n = 66)	65 ± 13	47 ± 6	21	0.19	[− 0.31, 0.83]	3.9 ± 0.2	3.8 ± 0.3	0.4	0.29	[− 0.35, 0.89]

*CMJ* countermovement jump, *SJ*: squat jump, *DJ*: drop jump, *DL* drop landing

#### Summary of statistical analysis

### Discussion

This pilot study is the first to evaluate the validity and reliability of the novel loadsol^®^ pro insoles for GRF measurements during landing tasks. Differences were noted between the recorded forces of insoles and force plate in some force–time graphs, indicating that the insoles generally underestimate the GRF (see Additional file 1: Figs. S2 and S5). Such an underestimation was also observed in another study with a pressure insole (Moticon Science Pro Sensor) as well [13]. There are many different studies, not specifically for gymnastics, that have reported mixed results [14–16].

Secondly, in the SPM plots, the data between x = 0 and x = 15 show a higher variation (see Additional file 1: Fig. S3). Consequently, the area around the maximal vertical GRF deviates because the maximal vertical GRF is at x = 9. Moreover, the maximal vertical GRF occurs within the first 0.1 s after touchdown of the foot, which would mean that the variation in the first milliseconds after landing is higher. This finding is in line with the literature, which suggests that errors occur mainly at the beginning of the touchdown of the foot [17]. Additionally, twelve SPM plots showed no significant difference in the force curves, which means that in 75% of the measurements the novel loadsol^®^ pro insoles measure the GRF well.

Concerning the validity, the RMSE values of the maximal vertical GRF are inferior to those of the impulse and they do not correlate with the mean force measured by the force plate. Furthermore, the lower limit of the ICC 95% confidence intervals was below 0.5 for all exercises (see Additional file 1: Tables S3 and S4). All p values (see Additional file 1: Table S3 and S4) were non-significant except for the impulse calculation of CMJ of Participant 2 (p = 0.01). Thus, the relationship between the novel loadsol^®^ pro insoles and force plates is poor and the insoles are not reliable when measuring the maximal vertical GRF or impulse of jumps. These findings are not consistent with the current literature on the novel loadsol^®^ pro insoles during declined, level and inclined walking and running [18]. In that paper, the validity of peak force and impulse was excellent and the ICC values were above 0.6, which indicates good to excellent reliability [18]. However, the categorisation scheme (> 0.75 excellent, 0.6–0.74 good, 0.4–0.59 fair and < 0.4 poor) for the ICC values was different and the peak force, respectively impulse were determined during walking and running, where the forces are generally lower than during landing tasks [18]. Therefore, it is difficult to directly compare the results of this paper with our findings. However, a possible reason for the poor results of our study could be the low sampling rate of the insole (200 Hz). This would lead to an inaccurate measurement of the maximal vertical GRF because the insoles could miss the maximal vertical GRF of a jump since it occurs in a short time period (approximately 0.002–0.004 s). This is supported by the Nyquist criterion, where the signal could be distorted if the sampling rate is too low. For the impulse calculation, the sampling rate has a lower impact because a longer time period is analysed, where 200 Hz is sufficient for an appropriate force recording for impulse calculation. Figure 2 illustrates this problem of a low sampling rate. With the limitations of the insole measurement and the fact that the use of force plates is difficult in the gymnastic setting, musculo-skeletal modeling and simulations might be an alternative option.

As seen in the Bland-Altmann diagrams, mean maximal vertical GRF below 50 N/kg has a deviation of less than 25 N/kg between the force plate and insoles; however, for a mean maximal vertical GRF above 50 N/kg, the differences are higher. Thus, the novel loadsol^®^ pro insole is only a useful analytical tool for GRF measurements up to 50 N/kg. Considering the literature, this appearance is common as the accuracy and precision of another insole vary across the level of applied pressure as well [19]. In the Bland-Altmann diagrams of impulse, the values vary little and across the whole range in a similar way. Hence, the novel loadsol^®^ pro insole and force plate measurements are comparable only when considering the impulse of a jump.

To conclude, the insoles are a valid measurement tool for GRF curve progression and impulse during landing but underestimate the maximal vertical GRF. However, not only the validity and reliability of a measurement device but also the practicality is important. The insoles are easy to use, portable, time-efficient and inexpensive compared to force plates.

### Limitations

One limitation of this study is the attachment of the insoles to the feet with elastic adhesive bandage, as normally the novel loadsol^®^ pro insoles are worn in shoes. Secondly, the small sample size would lead to a lack of generalisability of the data and therefore future studies with more athletes are needed. One more limitation are the dropouts of the insole measurements, which led to an exclusion of measurement runs. This may be improved by reducing interfering signals in the measurement room, or the force–time curves could be interpolated before evaluating the data. The here used landings after CMJ, SJ, DJ and DL are much less demanding comparted to a landing after a gymnastic jump. Lastly, a major limitation is the low sampling rate of the insole (200 Hz) since the force–time curve is less accurate and thus the maximal vertical GRF less precise. Consequently, in future high impact landing studies, the use of an insole with a higher sampling rate is suggested for a precise determination of the maximal vertical GRF.

## Supplementary Information

**Additional file 1: Figure S1** The novel loadsol^®^ pro insoles are attached to the foot with elastic adhesive bandage because gymnasts normally train barefoot and the measurements should be specific to artistic gymnastics. **Figure S2**. The force plate and insole measurements are labelled in blue respectively orange. a) participant 1, DJ 40cm; b) participant 1, DJ 60cm; c) participant 1, DL 20cm; d) participant 2, CMJ; e) participant 2, DJ 20cm; f) participant 2, DL 20cm CMJ: countermovement jump, DJ: drop jump, DL: drop landing. **Figure S3** SPM plots of all exercises of both participants (participant 1: a-h, 2: i-p). The x-axis is the time frame axis where the maximal vertical GRF is normally at x=9. a,i) CMJ; b,j) SJ; c,k) DJ 20cm; d,l) DJ 40cm; e,m) DJ 60cm; f,n) DL 20cm (in f the maximal vertical GRF is at x=6); g,o) DL 40cm (in g the maximal vertical GRF is at x=8); h,p) DL 60cm CMJ: countermovement jump, SJ: squat jump, DJ: drop jump, DL: drop landing. **Table S1** RMSE of maximal vertical GRF of different exercises of both participants CMJ: countermovement jump, SJ: squat jump, DJ: drop jump, DL: drop landing. **Table S2** RMSE of impulse of different exercises of both participants CMJ: countermovement jump, SJ: squat jump, DJ: drop jump, DL: drop landing. **Table S3** ICC values of maximal vertical GRF of the different exercises of both participants CMJ: countermovement jump, SJ: squat jump, DJ: drop jump, DL: drop landing. **Table S4** ICC values of impulse of the different exercises of both participants; * indicates significant correlation CMJ: countermovement jump, SJ: squat jump, DJ: drop jump, DL: drop landing. **Figure S4** Bland Altmann diagrams comparing force plate and insole measurements of maximal vertical GRF (a, c) and impulse (b, d) of both participants. For the difference the insole values are subtracted from the force plate values. The mean of all values is displayed in green and the limits of agreement are labelled in grey. **Figure S5** Bland Altmann diagram comparing force plate and insole measurements of maximal vertical GRF of both participants (n=66). For the difference the insole values are subtracted from the force plate values. The mean of all values is displayed in green and the limits of agreement are labelled in grey. Generally, the insoles underestimate the GRF. Therefore, a formula was developed for a force range of 28.5 to 90N/kg to calculate the real GRF value: Real GRF value in N/kg = 28.5 + (measured GRF value in N/kg – 28.5) * 1.65.

## Data Availability

All the data is available on a repository with the https://doi.org/10.6084/m9.figshare.21316410.

## Abbreviations

GRF: Ground reaction force
SFISM: Swiss Federal Institute of Sport Magglingen
CMJ: Countermovement jump
SJ: Squat jump
DJ: Drop jump
DL: Drop landing
SPM: Statistical parametric mapping
RMSE: Root mean square error
ICC: Intraclass correlation coefficient
SD: Standard deviation

## References

[1] Wolf SF, LaBella CR (2020). Epidemiology of gymnastics injuries. Gymnast Med Eval Manag Rehabil..

[2] Sweeney EA, Howell DR, James DA, Potter MN, Provance AJ (2018). Returning to sport after gymnastics injuries. Curr Sports Med Rep..

[3] Edouard P, Steffen K, Junge A, Leglise M, Soligard T, Engebretsen L (2018). Gymnastics injury incidence during the 2008, 2012 and 2016 Olympic Games: analysis of prospectively collected surveillance data from 963 registered gymnasts during Olympic Games. Br J Sports Med.

[4] Straker R, Exell TA, Farana R, Hamill J, Irwin G (2021). Biomechanical responses to landing strategies of female artistic gymnasts. Eur J Sport Sci.

[5] Glynn B, Laird J, Herrington L, Rushton A, Heneghan NR (2022). Analysis of landing performance and ankle injury in elite British artistic gymnastics using a modified drop land task: a longitudinal observational study. Phys Ther Sport.

[6] List R, Hitz M, Angst M, Taylor WR, Lorenzetti S (2017). In-situ force plate calibration: 12 years’ experience with an approach for correcting the point of force application. Gait Posture.

[7] Simons C, Bradshaw EJ (2016). Reliability of accelerometry to assess impact loads of jumping and landing tasks. Sports Biomech.

[8] Pérez-Soriano P, Llana-Belloch S, Morey-Klapsing G, Perez-Turpin JA, Cortell-Tormo JM, van den Tillaar R (2010). Effects of mat characteristics on plantar pressure patterns and perceived mat properties during landing in gymnastics. Sports Biomech.

[9] Pataky TC, Vanrenterghem J, Robinson MA (2016). The probability of false positives in zero-dimensional analyses of one-dimensional kinematic, force and EMG trajectories. J Biomech.

[10] Chai T, Draxler RR (2014). Root mean square error (RMSE) or mean absolute error (MAE)?—arguments against avoiding RMSE in the literature. Geosci Model Dev.

[11] Koo TK, Li MY (2016). A guideline of selecting and reporting intraclass correlation coefficients for reliability research. J Chiropr Med.

[12] Martin Bland J, Altman DG (1986). Statistical methods for assessing agreement between two methods of clinical measurement. Lancet.

[13] Löfquist I, Björklund G (2020). What magnitude of force is a slopestyle Skier exposed to when landing a big air jump?. Int J Exerc Sci.

[14] DeBerardinis J, Dufek JS, Trabia MB, Lidstone DE (2018). Assessing the validity of pressure-measuring insoles in quantifying gait variables. J Rehabil Assist Technol Eng..

[15] Koch M, Lunde LK, Ernst M, Knardahl S, Veiersted KB (2016). Validity and reliability of pressure-measurement insoles for vertical ground reaction force assessment in field situations. Appl Ergon.

[16] Giacomozzi C (2010). Appropriateness of plantar pressure measurement devices: a comparative technical assessment. Gait Posture.

[17] Forner Cordero A, Koopman HJFM, Van Der Helm FCT (2004). Use of pressure insoles to calculate the complete ground reaction forces. J Biomech.

[18] Renner KE, Blaise Williams DS, Queen RM (2019). The reliability and validity of the Loadsol^®^ under various walking and running conditions. Sensors.

[19] Hsiao H, Guan J, Weatherly M (2010). Accuracy and precision of two in-shoe pressure measurement systems. Ergonomics.

