# Supplementary material for: Pilot study: validity and reliability of textile insoles used to measure the characteristics of landing tasks during rehabilitation and artistic gymnastics
Source: BMC Res Notes. 2023 Apr 22;16:59. doi: 10.1186/s13104-023-06328-9 (PMC10122392; doi:10.1186/s13104-023-06328-9)
Supplement: Supplementary file 1 — Additional file 1: Figure S1 The novel loadsol® pro insoles are attached to the foot with elastic adhesive bandage because gymnasts normally train barefoot and the measurements should be specific to artistic gymnastics. Figure S2. The force plate and insole measurements are labelled in blue respectively orange. a) participant 1, DJ 40cm; b) participant 1, DJ 60cm; c) participant 1, DL 20cm; d) participant 2, CMJ; e) participant 2, DJ 20cm; f) participant 2, DL 20cm CMJ: countermovement jump, DJ: drop jump, DL: drop landing. Figure S3 SPM plots of all exercises of both participants (participant 1: a-h, 2: i-p). The x-axis is the time frame axis where the maximal vertical GRF is normally at x=9. a,i) CMJ; b,j) SJ; c,k) DJ 20cm; d,l) DJ 40cm; e,m) DJ 60cm; f,n) DL 20cm (in f the maximal vertical GRF is at x=6); g,o) DL 40cm (in g the maximal vertical GRF is at x=8); h,p) DL 60cm CMJ: countermovement jump, SJ: squat jump, DJ: drop jump, DL: drop landing. Table S1 RMSE of maximal vertical GRF of different exercises of both participants CMJ: countermovement jump, SJ: squat jump, DJ: drop jump, DL: drop landing. Table S2 RMSE of impulse of different exercises of both participants CMJ: countermovement jump, SJ: squat jump, DJ: drop jump, DL: drop landing. Table S3 ICC values of maximal vertical GRF of the different exercises of both participants CMJ: countermovement jump, SJ: squat jump, DJ: drop jump, DL: drop landing. Table S4 ICC values of impulse of the different exercises of both participants; * indicates significant correlation CMJ: countermovement jump, SJ: squat jump, DJ: drop jump, DL: drop landing. Figure S4 Bland Altmann diagrams comparing force plate and insole measurements of maximal vertical GRF (a, c) and impulse (b, d) of both participants. For the difference the insole values are subtracted from the force plate values. The mean of all values is displayed in green and the limits of agreement are labelled in grey. Figure S5 Bland Altmann diagram comparing for [file 13104_2023_6328_MOESM1_ESM.docx]

**Additional file 1 to “Pilot study: validity and reliability of textile insoles used to measure the characteristics of landing tasks during rehabilitation and artistic gymnastics”**

**
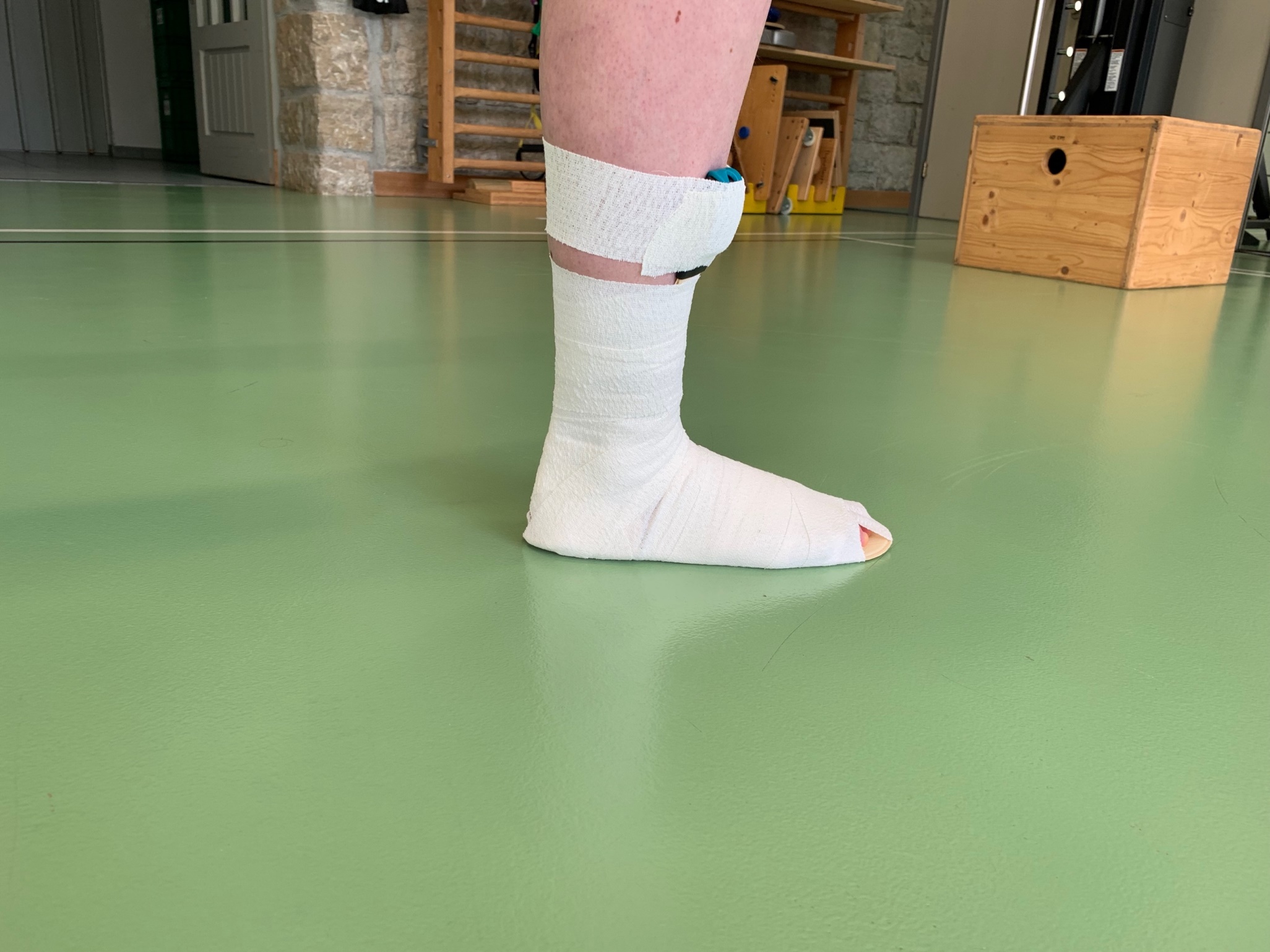
Insole attachment**

Figure S1 The novel loadsol^®^ pro insoles are attached to the foot with elastic adhesive bandage because gymnasts normally train barefoot and the measurements should be specific to artistic gymnastics.

**
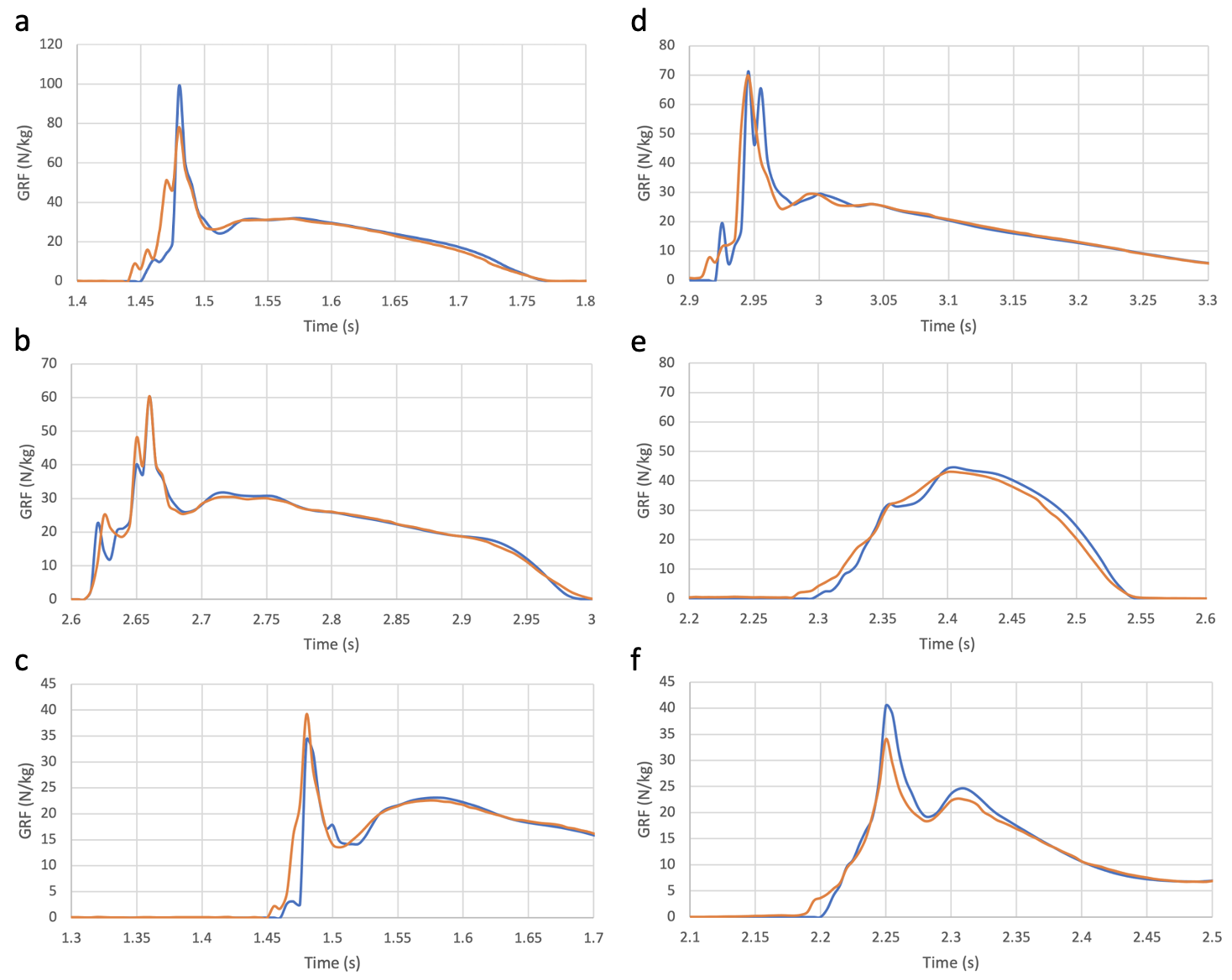
Ground reaction force (GRF) of different jumps**

Figure S2 Ground reaction force (GRF) of different jumps. The force plate and insole measurements are labelled in blue respectively orange. a) participant 1, DJ 40cm; b) participant 1, DJ 60cm; c) participant 1, DL 20cm; d) participant 2, CMJ; e) participant 2, DJ 20cm; f) participant 2, DL 20cm
CMJ: countermovement jump, DJ: drop jump, DL: drop landing

**
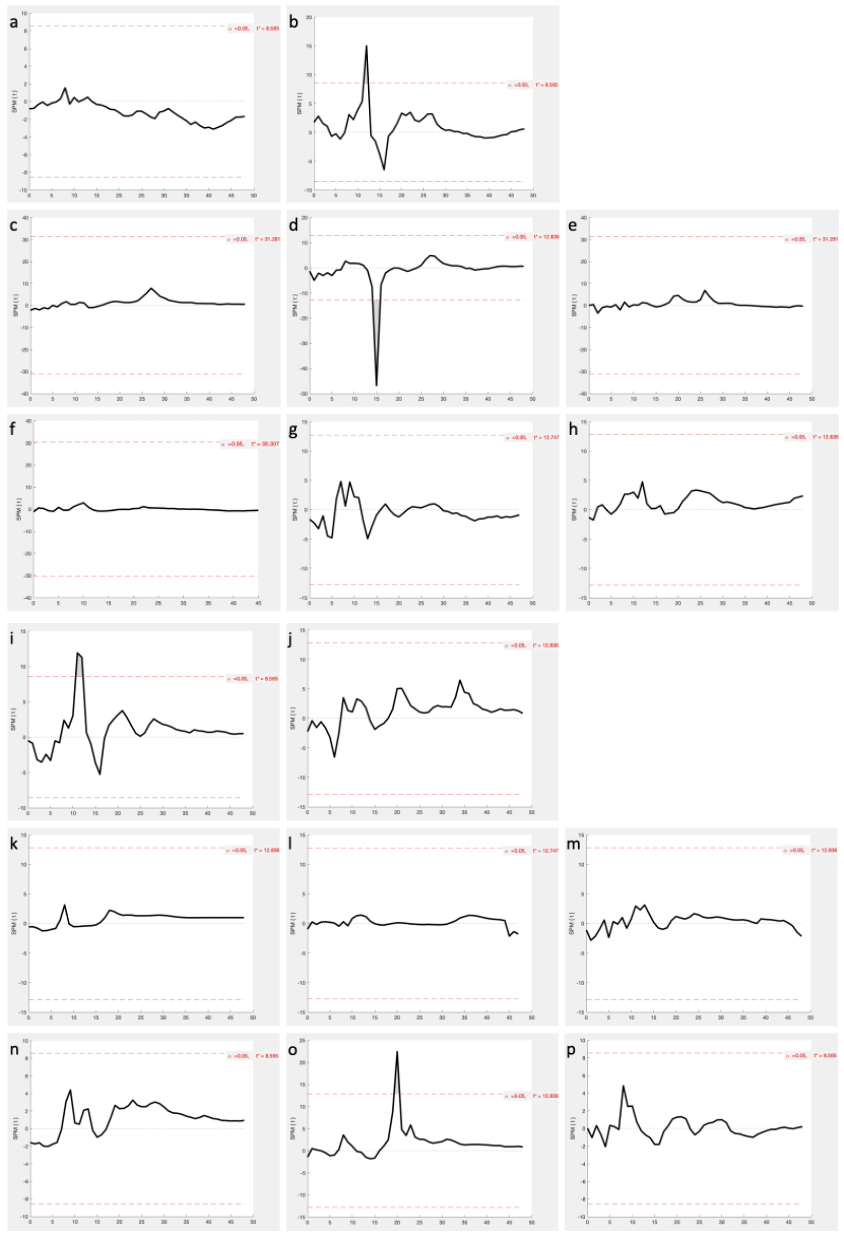
SPM plots of both participants**

Figure S3 SPM plots of all exercises of both participants (participant 1: a-h, 2: i-p). The x-axis is the time frame axis where the maximal vertical GRF is normally at x=9.
a,i) CMJ; b,j) SJ; c,k) DJ 20cm; d,l) DJ 40cm; e,m) DJ 60cm; f,n) DL 20cm (in f the maximal vertical GRF is at x=6); g,o) DL 40cm (in g the maximal vertical GRF is at x=8); h,p) DL 60cm
CMJ: countermovement jump, SJ: squat jump, DJ: drop jump, DL: drop landing

**RMSE of maximal vertical GRF**

| participant 1 | | | | |
| --- | --- | --- | --- | --- |
| **Exercise** | **∅ force plate (N/kg) and SD** | **∅ insole (N/kg) and SD** | **RMSE (N/kg)** | **% of mean force (force plate)** |
| CMJ | 43 ± 13 | 33 ± 1 | 16 | 37 |
| SJ | 48 ± 9 | 34 ± 2 | 17 | 36 |
| DJ 20cm | 50 ± 7 | 39 ± 2 | 14 | 28 |
| DJ 40cm | 69 ± 22 | 52 ± 16 | 20 | 30 |
| DJ 60cm | 78 ± 14 | 60 ± 4 | 26 | 33 |
| DL 20cm | 37 ± 4 | 33 ± 5 | 8 | 22 |
| DL 40cm | 71 ± 9 | 44 ± 2 | 28 | 40 |
| DL 60cm | 87 ± 34 | 45 ± 9 | 50 | 57 |
| **Mean (n=31)** | **60 ± 14** | **43 ± 5** | **22** | **35** |
| participant 2 | | | | |
| CMJ | 65 ± 13 | 52 ± 10 | 17 | 26 |
| SJ | 88 ± 19 | 53 ± 4 | 39 | 44 |
| DJ 20cm | 47 ± 3 | 43 ± 3 | 5 | 10 |
| DJ 40cm | 53 ± 3 | 53 ± 2 | 2 | 3 |
| DJ 60cm | 78 ± 4 | 73 ± 12 | 11 | 14 |
| DL 20cm | 57 ± 16 | 36 ± 6 | 24 | 43 |
| DL 40cm | 87 ± 22 | 47 ± 5 | 44 | 51 |
| DL 60cm | 74 ± 11 | 54 ± 10 | 21 | 29 |
| **Mean (n=35)** | **69 ± 11** | **51 ± 6** | **20** | **27** |
| **Mean (n=66)** | **65 ± 13** | **47 ± 6** | **21** | **31** |

Table S1 RMSE of maximal vertical GRF of different exercises of both participants
CMJ: countermovement jump, SJ: squat jump, DJ: drop jump, DL: drop landing

**RMSE of impulse**

| participant 1 | | | | |
| --- | --- | --- | --- | --- |
| **Exercise** | **∅ force plate (Ns/kg) and SD** | **∅ insole (Ns/kg) and SD** | **RMSE (Ns/kg)** | **% of mean impulse (force plate)** |
| CMJ | 3.3 ± 0.2 | 3.5 ± 0.4 | 0.3 | 9.2 |
| SJ | 3.0 ± 0.1 | 3.0 ± 0.1 | 0.2 | 6.3 |
| DJ 20cm | 4.4 ± 0.1 | 4.3 ± 0.03 | 0.2 | 3.9 |
| DJ 40cm | 4.7 ± 0.1 | 4.8 ± 0.1 | 0.1 | 2.3 |
| DJ 60cm | 5.3 ± 0.2 | 5.2 ± 0.1 | 0.1 | 2.7 |
| DL 20cm | 2.4 ± 0.1 | 2.6 ± 0.3 | 0.4 | 18.2 |
| DL 40cm | 3.2 ± 0.1 | 3.9 ± 1.2 | 1.4 | 42.9 |
| DL 60cm | 3.7 ± 0.1 | 3.3 ± 0.1 | 0.4 | 10.4 |
| **Mean (n=31)** | **3.8 ± 0.1** | **3.8 ± 0.3** | **0.4** | **12.0** |
| participant 2 | | | | |
| CMJ | 3.6 ± 0.2 | 3.7 ± 0.4 | 0.2 | 4.6 |
| SJ | 3.6 ± 0.2 | 3.6 ± 0.1 | 0.1 | 3.9 |
| DJ 20cm | 4.4 ± 0.1 | 4.0 ± 0.2 | 0.4 | 9.8 |
| DJ 40cm | 5.2 ± 0.1 | 4.9 ± 0.1 | 0.3 | 5.4 |
| DJ 60cm | 5.8 ± 0.2 | 5.7 ± 0.2 | 0.2 | 3.2 |
| DL 20cm | 2.0 ± 0.2 | 1.7 ± 0.3 | 0.4 | 20.6 |
| DL 40cm | 3.4 ± 0.1 | 3.1 ± 0.2 | 0.4 | 11.3 |
| DL 60cm | 4.0 ± 0.1 | 3.7 ± 0.4 | 0.5 | 12.2 |
| **Mean (n=35)** | **4.0 ± 0.2** | **3.8 ± 0.2** | **0.3** | **8.9** |
| **Mean (n=66)** | **3.9 ± 0.2** | **3.8 ± 0.3** | **0.4** | **10.5** |

Table S2 RMSE of impulse of different exercises of both participants
CMJ: countermovement jump, SJ: squat jump, DJ: drop jump, DL: drop landing

**ICC and p-values of maximal vertical GRF**

| participant 1 | | | | |
| --- | --- | --- | --- | --- |
| **Exercise** | **ICC value** | **p-value** | **95% confidence interval** | |
| CMJ | 0.04 | 0.46 | -0.52 | 0.79 |
| SJ | 0.02 | 0.46 | -0.19 | 0.63 |
| DJ 20cm | -0.22 | 0.81 | -0.42 | 0.80 |
| DJ 40cm | 0.66 | 0.07 | -0.15 | 0.97 |
| DJ 60cm | -0.20 | 0.72 | -0.53 | 0.87 |
| DL 20cm | -0.16 | 0.59 | -1.20 | 0.94 |
| DL 40cm | -0.01 | 0.57 | -0.08 | 0.49 |
| DL 60cm | 0.20 | 0.24 | -0.19 | 0.87 |
| **Mean (n=31)** | **0.04** | **0.49** | **-0.41** | **0.79** |
| participant 2 | | | | |
| CMJ | 0.41 | 0.12 | -0.20 | 0.90 |
| SJ | 0.07 | 0.33 | -0.12 | 0.75 |
| DJ 20cm | 0.43 | 0.13 | -0.14 | 0.94 |
| DJ 40cm | 0.86 | 0.04 | -0.21 | 0.99 |
| DJ 60cm | 0.46 | 0.19 | -0.59 | 0.95 |
| DL 20cm | 0.20 | 0.22 | -0.17 | 0.80 |
| DL 40cm | 0.08 | 0.31 | -0.12 | 0.76 |
| DL 60cm | 0.29 | 0.16 | -0.09 | 0.84 |
| **Mean (n=35)** | **0.35** | **0.19** | **-0.21** | **0.87** |
| **Mean (n=66)** | **0.19** | **0.34** | **-0.31** | **0.83** |

Table S3 ICC values of maximal vertical GRF of the different exercises of both participants
CMJ: countermovement jump, SJ: squat jump, DJ: drop jump, DL: drop landing

**ICC and p-values of impulse**

| participant 1 | | | | |
| --- | --- | --- | --- | --- |
| **Exercise** | **ICC value** | **p-value** | **95% confidence interval** | |
| CMJ | 0.63 | 0.06 | -0.17 | 0.95 |
| SJ | -0.09 | 0.58 | -0.84 | 0.78 |
| DJ 20cm | 0.25 | 0.22 | -0.19 | 0.95 |
| DJ 40cm | 0.68 | 0.06 | -0.16 | 0.97 |
| DJ 60cm | 0.74 | 0.06 | -0.21 | 0.99 |
| DL 20cm | -1.07 | 0.82 | -2.11 | 0.85 |
| DL 40cm | 0.004 | 0.5 | -0.86 | 0.88 |
| DL 60cm | 0.13 | 0.2 | -0.04 | 0.76 |
| **Mean (n=31)** | **0.16** | **0.31** | **-0.57** | **0.89** |
| participant 2 | | | | |
| CMJ | 0.89 | 0.01* | 0.26 | 0.99 |
| SJ | 0.68 | 0.07 | -0.3 | 0.98 |
| DJ 20cm | 0.16 | 0.22 | -0.1 | 0.82 |
| DJ 40cm | 0.1 | 0.26 | -0.08 | 0.74 |
| DJ 60cm | 0.72 | 0.06 | -0.12 | 0.98 |
| DL 20cm | 0.39 | 0.13 | -0.15 | 0.89 |
| DL 40cm | 0.24 | 0.19 | -0.09 | 0.87 |
| DL 60cm | 0.11 | 0.37 | -0.39 | 0.8 |
| **Mean (n=35)** | **0.41** | **0.16** | **-0.12** | **0.88** |
| **Mean (n=66)** | **0.29** | **0.24** | **-0.35** | **0.89** |

Table S4 ICC values of impulse of the different exercises of both participants; * indicates significant correlation
CMJ: countermovement jump, SJ: squat jump, DJ: drop jump, DL: drop landing

**
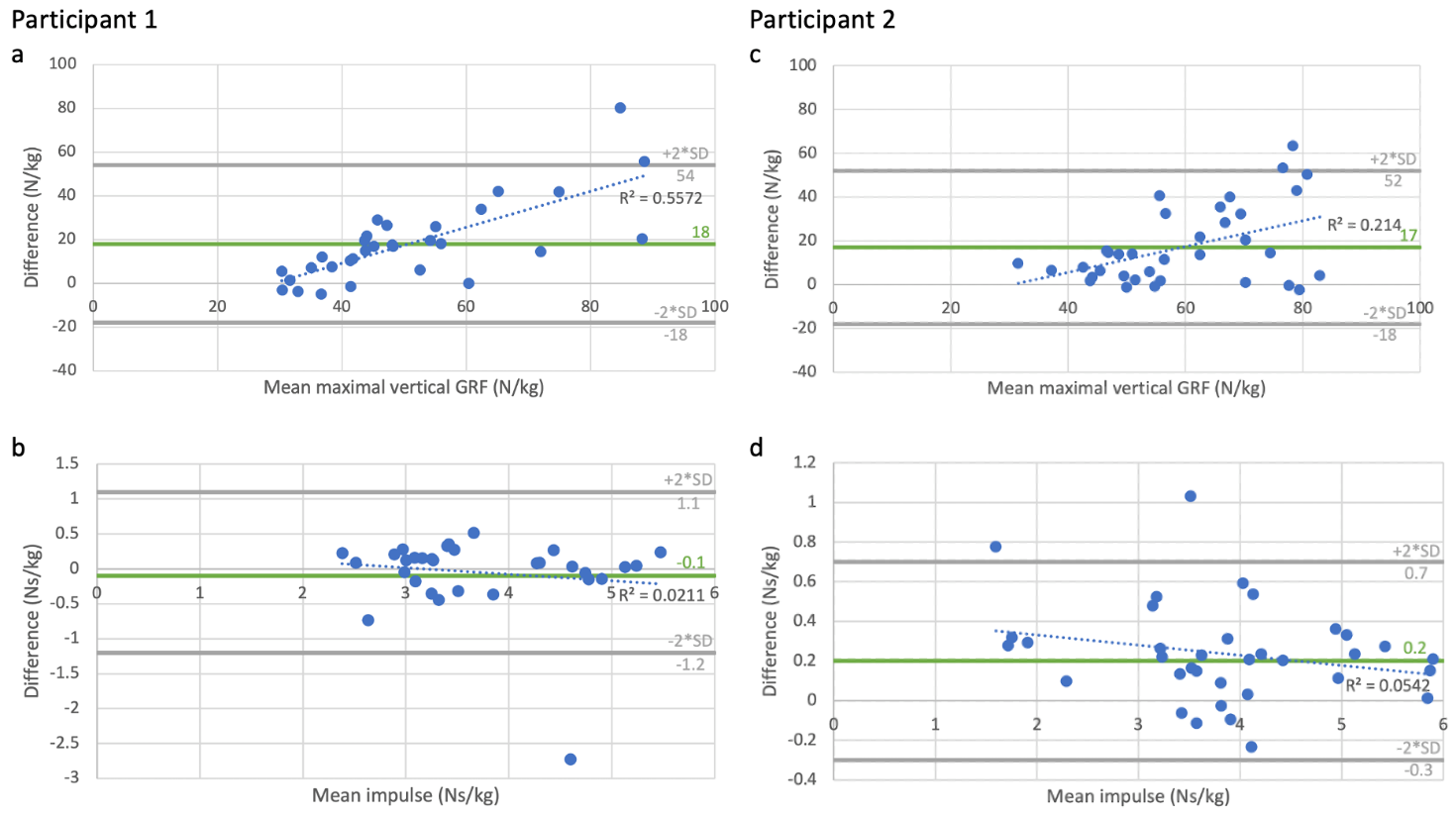
Bland-Altmann diagrams**

Figure S4 Bland Altmann diagrams comparing force plate and insole measurements of maximal vertical GRF (a, c) and impulse (b, d) of both participants. For the difference the insole values are subtracted from the force plate values. The mean of all values is displayed in green and the limits of agreement are labelled in grey.

**
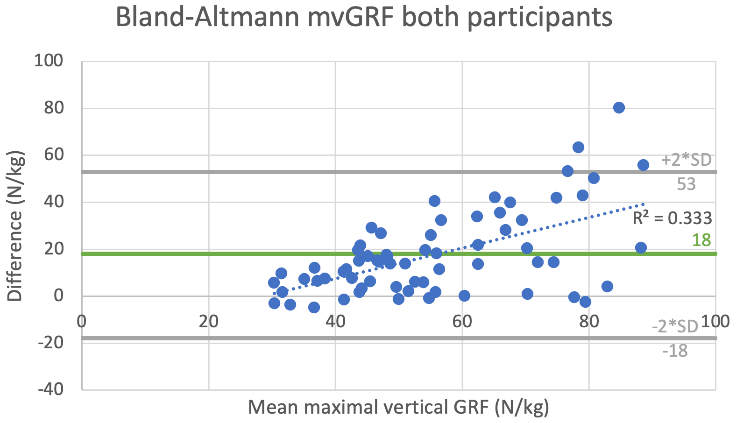
Bland-Altmann diagram of maximal vertical GRF of both participants**

Figure S5 Bland Altmann diagram comparing force plate and insole measurements of maximal vertical GRF of both participants (n=66). For the difference the insole values are subtracted from the force plate values. The mean of all values is displayed in green and the limits of agreement are labelled in grey.
Generally, the insoles underestimate the GRF. Therefore, a formula was developed for a force range of 28.5 to 90N/kg to calculate the real GRF value:
Real GRF value in N/kg = 28.5 + (measured GRF value in N/kg – 28.5) * 1.65
